# Dynamic Compressive Behavior, Constitutive Modeling, and Complete Failure Criterion of 30 Vol.% B_4_C/2024Al Composite

**DOI:** 10.3390/ma18051170

**Published:** 2025-03-06

**Authors:** Qiang Yan, Zhihong Zhao, Tian Luo, Feng Li, Jianjun Zhao, Zhenlong Chao, Sanfeng Liu, Yong Mei, Fengjun Zhou

**Affiliations:** 1 Institute of Defense Engineering, Academy of Military Science, People’s Liberation Army, Beijing 100036, China; yanqiang950131@163.com (Q.Y.);; 2 School of Materials Science and Engineering, Harbin Institute of Technology, Harbin 150001, China; 3 Chinese Academy of Engineering, Beijing 100088, China

**Keywords:** aluminum matrix composites, dynamic compression, constitutive model, complete failure criterion, finite element simulation

## Abstract

This study investigated the compressive behavior of 30 vol.% boron carbide (B_4_C)/2024 aluminum (Al) composites under quasi-static and dynamic loading at different temperatures. Building on the experimental findings, the Johnson–Cook (JC) model was modified, and a complete failure criterion was proposed. These were validated in Abaqus employing the user subroutine for hardening (VUHARD), which incorporated both the modified JC (MJC) model and the complete failure criterion. Experimental results revealed that strain softening was an important feature of the stress–strain curve. The analysis of mechanisms contributing to yield strength revealed that Taylor and load transfer mechanisms dominated, accounting for 89.6% of the total enhancement. Microstructural analysis identified particle fracture and matrix damage were the primary mechanisms driving material failure. Microcracks mainly propagated through the matrix and interface or directly through the ceramic particles and the matrix. The MJC model demonstrated high accuracy in describing the plastic deformation behavior of the composite, with a mean absolute error (MAE) below 15% under dynamic loading. Further simulation confirmed that finite element analyses using the VUHARD subroutine accurately captured the plastic deformation and crack propagation behaviors of the composite under dynamic loading. This study offers a novel approach to describe the plastic deformation and failure behaviors of ceramic-reinforced aluminum matrix composites under dynamic loading conditions.

## 1. Introduction

Aluminum matrix composites (AMCs) are widely used in the impact protection field due to their high specific strength and stiffness [1,2,3,4,5]. In recent years, significant advancements have been made in AMCs, including the development of biomimetic armor and functionally graded materials (FGMs), which have demonstrated remarkable effectiveness in protective performance [6,7,8,9]. Among these, boron carbide (B_4_C)/aluminum (Al) composites are commonly used, as B_4_C ceramic particles have high hardness (~38 GPa) and a density (2.52 g/cm^3^) comparable to that of the Al matrix (2.7 g/cm^3^). Research has demonstrated a strong correlation between dynamic compression properties and impact resistance, and improved compression behavior could enhance impact protection capabilities [10,11]. Therefore, understanding the dynamic compression behavior of B_4_C/Al composites and developing a constitutive model for dynamic loading conditions are crucial for advancing their application in impact protection.

Liu Bin et al. [12] conducted dynamic compression tests on 55 vol.% B_4_C/Al composite with different particle sizes and found that the composite containing 3 μm particles exhibited superior strength, which consistently increased with impact velocity. In contrast, the composite with 20 μm particles exhibited a reduction when the impact velocity increased from 28 m/s to 33 m/s. S.J. Ye et al. [13] investigated the quasi-static and dynamic compression behavior of 15 wt.% B_4_C/Al composite. By utilizing digital image correlation (DIC), it was found that the dynamic strain fields were more homogeneous compared to quasi-static conditions, which contributes to a higher density of geometrically necessary dislocations and results in significant strain hardening and strain rate strengthening. Wang Yang et al. [11,14] revealed that within a B_4_C content range of 20–40 vol.%, the composites exhibited greater compressive strength under dynamic conditions compared to quasi-static loading, with pronounced strain hardening, strain softening, and strain rate strengthening behaviors, indicating that the strain softening was driven by adiabatic shear. An increase in B_4_C content enhanced both composite strength and strain rate sensitivity while altering damage mechanisms. Furthermore, studies on a 16 vol.% B_4_C/2024Al composite indicated that elevated temperatures exert significant softening behavior and affect damage modes and mechanisms [15]. Based on these studies, B_4_C/Al composites demonstrated strain hardening, strain rate strengthening, and strain softening behaviors. Material composition and loading conditions significantly influence these behaviors and damage development. However, systematic research on the dynamic compression behavior of B_4_C/Al composites remains limited.

Given the limited research on AMCs, this review has been expanded to include the constitutive models of metal matrix composites (MMCs) under dynamic loading. Foundational constitutive models are categorized into physically based models, such as the Zerilli–Armstrong (Z-A) model, phenomenological models like the Johnson–Cook (JC) model, and hybrid models, including the JC-ZA and Arrhenius models, which combine both approaches [16,17,18,19]. Recent studies have modified models by incorporating microscopic damage mechanisms of MMCs. M. E. Naguib et al. [20] considered three types of microscopic damage and developed an integrated numerical model. This model employed the Gurson–Tvergaard–Needleman (GTN) model to describe void growth and coalescence in the matrix, the Johnson–Holmquist II (JH-2) model to represent ceramic particle fragmentation, and the cohesive zone method to characterize particle–matrix debonding. Similarly, H. Ban et al. [21] incorporated microscopic damage into a nonlinear elastoplastic constitutive relationship. This was achieved by applying the secant modulus method, low-order strain gradient theory under damage, and effective modulus reduction techniques. Based on the JC model, A. Ghandehariun et al. [22] developed separate flow stress equations for undamaged materials, particle fragmentation, and interfacial debonding. These equations were combined using volume fraction and Weibull’s weakest link theory through linear combination to derive a constitutive equation describing macroscopic deformation in shear zones. Recent research has focused on incorporating damage mechanisms into constitutive models. However, studies on AMCs remain limited, particularly regarding the modification of phenomenological models.

This study investigates a 30 vol.% B_4_C/2024Al composite to systematically examine the effects of strain rate and elevated temperature on its compressive behavior under both quasi-static and dynamic loading conditions, focusing on stress–strain curves, strengthening mechanisms, and damage mechanisms. Based on the characteristics of stress–strain curves, the JC model will be modified, and a complete failure criterion based on strain energy density *W_D_* will be proposed. Subsequently, the model and criterion will be further developed into the VUHARD subroutine and validated through ABAQUS simulations of the split Hopkinson pressure bar (SHPB).

## 2. Materials and Methods

### 2.1. The 30 Vol.%B_4_C/2024Al Composite

**Figure 1:**
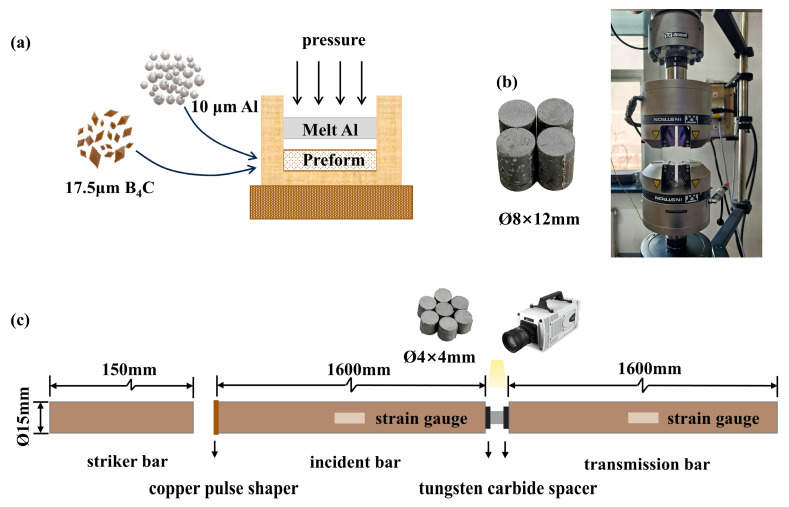
Fabrication, Processing, and Compression Testing of 30 vol.% B_4_C/2024Al Composite. (**a**) Fabrication through the pressure infiltration process. (**b**) Schematic of the quasi-static compression test configuration. (**c**) Schematic of the SHPB test configuration.

The 30 vol.% B_4_C/2024Al composite contains B_4_C particles (Mudanjiang Jingang Diamond Boron Carbide Co., Ltd., Mudanjiang, China) with an average diameter of 17.5 μm. To achieve the desired B_4_C volume fraction, aluminum powder (Northeast Light Alloy Co., Ltd., Harbin, China) with a diameter of 10 μm was incorporated into the mixture. The volume ratio of aluminum powder to B_4_C is 0.84. The composite was fabricated using a pressure infiltration process, as shown in Figure 1a. First, the powder mixture was placed into a 90 mm mold and subjected to cold pressing to form a preform. Subsequently, molten 2024Al (Northeast Light Alloy Co., Ltd., Harbin, China) was introduced into the mold and pressurized until complete solidification was achieved. After fabrication, the composites underwent T6 heat treatment, consisting of solution treatment at 495 °C for 1 h, followed by quenching and aging at 190 °C for 8 h.

### 2.2. Quasi-Static and Dynamic Compression Test

The quasi-static compression performance was tested using a universal testing machine at a loading rate of 0.5 mm/min, with sample dimensions of Ø8 mm × 12 mm (Figure 1b). The dynamic compression performance was tested using a SHPB, with sample dimensions of Ø4 mm × 4 mm. In the SHPB apparatus, the striker bar is 250 mm long and the incident and transmission bars are each 1600 mm long, with all bars having a diameter of 15.0 mm. A high-speed camera was aligned with the sample, the SHPB testing configuration is illustrated in Figure 1c. Before testing, all samples were prepared to ensure flatness at both ends and polished with diamond grinding papers ranging from 400 to 2000 grit to remove the wire-cutting marks. For high-temperature tests, an electromagnetic heating furnace was used to heat the samples to the target temperature which was maintained for 15 min before loading. After testing, the samples were immediately immersed in cold water to preserve their microstructural integrity.

The target strain rate for the quasi-static tests was 0.01/s, while for the dynamic tests, strain rates of 2000/s, 4000/s, and 6000/s were used. Tests were conducted at 298.15 K, 523.15 K, and 723.15 K. To ensure data accuracy, at least five samples were tested for each condition.

### 2.3. Microstructural Characterization

The microstructural characterization of the polished samples and the internal damage features after compression testing were investigated using a scanning electron microscope (SEM, Zeiss Supra55, Jena, Germany). The SEM was operated in secondary electron mode (SE) with an accelerating voltage of 20.00 kV.

### 2.4. VUHARD Subroutine Implementation

**Figure 2:**
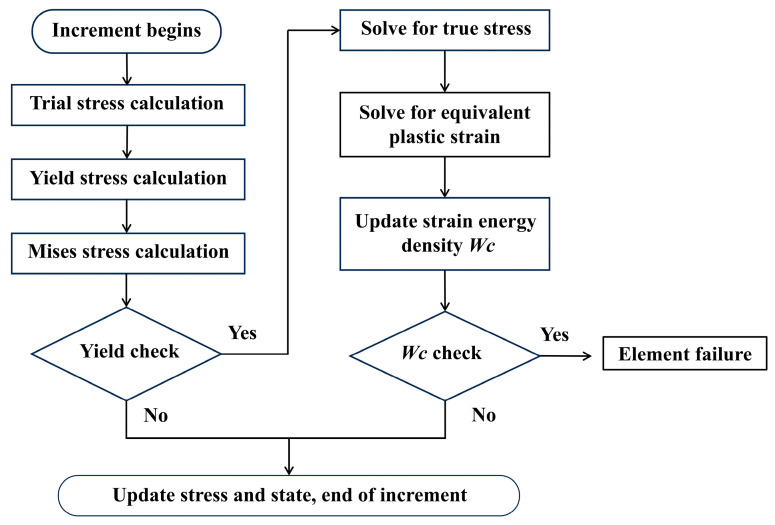
Flowchart of VUHARD subroutine development.

To implement the MJC model and complete failure criterion, the VUHARD subroutine in ABAQUS for explicit dynamic analysis was required. The implementation of the VUHARD subroutine followed a sequential workflow, which included the initiation of incremental steps, trial stress calculation, MJC yield stress calculation, yield determination, stress updating, *W_D_* calculation, cumulative strain energy density *W_C_* updating, complete failure determination, and the finalization of the incremental step, as illustrated in Figure 2. Yield determination followed the Mises yield criterion, where the trial stress was compared with the yield stress obtained from the MJC model to determine whether yielding occurred. Stress was updated using the radial return mapping (RMP) method, which projects stress onto the yield surface using the plastic flow rule and consistency condition when the stress state exceeds the yield surface. The nonlinear equations in this procedure were solved using the Newton–Raphson iterative method, ensuring convergence and accuracy. The cumulative strain energy density at the current time step was updated by adding the product of the equivalent plastic strain rate, yield stress, and the time step (Δ*t*) to the value from the previous time step.

### 2.5. Numerical Simulation

A full-scale SHTB finite element model was created using ABAQUS/Explicit, based on the experimental setup described in Section 2.2. The material properties of the incident and transmission bars were defined by their density and modulus, with values of 7.85 × 10⁻^9^ and 2.10 × 10^5^. The mesh element type was set to C3D8R with a global mesh size of 0.05. Surface-to-surface contact was established between the internal nodes of the specimen mesh and the end faces of the two bars. Loading was applied at the end face of the incident bar based on the incident stress–time curve from the experiment. The Fortran-based VUHARD subroutine was integrated into the job setup. Validation was conducted for strain rates of 2000/s, 4000/s, and 6000/s.

## 3. Experimental Results and Discussion

**Figure 3:**
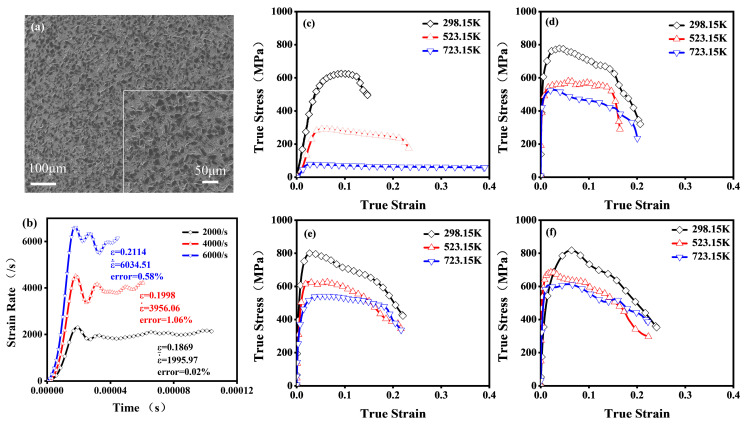
Processed samples and Compression test results. (**a**) Low- and high-resolution SEM images of the processed samples. (**b**) Strain rate versus time curves in SHPB testing at 298.15 K. (**c**–**f**) True stress–strain curves at strain rates of 0.01/s, 2000/s, 4000/s, and 6000/s.

Figure 3a presents SEM images of the polished surface of the samples. These images reveal a continuous aluminum matrix with uniformly dispersed ceramic particles and no significant defects, ensuring the reliability and repeatability of the 30 vol.% B_4_C/2024Al composite in compression testing.

### 3.1. Stress–Strain Curve

To validate the reliability of SHPB testing, strain rate–time relationships were measured under high strain rate conditions at 298.15 K. As shown in Figure 3b, the curves under different strain rates exhibited an initial rapid increase, followed by a transition to a stable phase. Analysis of the stable phase confirmed that the average measured strain rate deviated by less than 1% from the target strain rate, indicating that the tests met the design requirements.

As shown in Figure 3c–f, the true stress–strain curves under various testing conditions consistently presented linear elastic, strain hardening, and strain softening stages. Analysis of these curves revealed that the composite exhibited distinct behaviors compared to the matrix. At 298.15 K and 0.01/s, it showed a higher elastic modulus and yield strength in the linear elastic stage. During the early plastic stage, it underwent a faster strain-hardening stage, reaching a quasi-static compressive strength of 628.1 MPa, greater than the matrix’s. The hardening stage transitioned into strain softening, where the stress decreased progressively with increasing strain. Ultimately, its failure strain was significantly lower than the matrix’s [23].

With an increasing strain rate, the composite showed a significant strain rate strengthening behavior. In the early plastic stage, the yield strength and flow stresses increased, the hardening process accelerated, and the compressive strength rose markedly from 628.1 MPa at 0.01/s to 834.8 MPa at 6000/s. At higher strain rates, the onset of the softening stage shifted to an earlier point, and the slope of the softening stage became steeper, reflecting an enhanced strain softening effect. As shown in Figure 3c–f at 298.15 K, increasing the strain rate from 0.01/s to 2000/s allowed the composite to deform more before fracture, absorbing more impact energy and increasing fracture strain. However, from 2000/s to 6000/s, this enhancement effect became less significant. Increasing temperature caused the yield stress and flow stress to decrease due to temperature softening. This effect was significant at a strain rate of 0.01/s but diminished at higher strain rates (2000/s–6000/s), possibly because dislocation movement was restricted at higher strain rates, preventing the full development of the temperature softening process. This suggests a coupling effect between temperature softening and strain rate strengthening on flow stress.

### 3.2. Strengthening Mechanisms

The yield strength of the 30 vol.% B_4_C/2024Al composite was significantly higher compared with 2024Al. Previous studies suggested that the strength enhancement in particle-reinforced MMCs is primarily attributed to the Orowan strengthening mechanism, Hall–Petch mechanism, Taylor strengthening mechanism, and load transfer mechanism [1,24,25,26]. The corresponding expressions for each mechanism are provided below.

Orowan strengthening $\Delta \sigma_{Or\mathrm{owan}}$ [27]:

(1) $$\Delta \sigma_{Or\mathrm{owan}}=\frac{\varphi G_{m}b}{d}{\left(\frac{6f}{\pi }\right)}^{1/3}$$

φ is a constant, given as 2 [27]. *Gm* denotes the shear modulus of the 2024Al, valued at 2.7 × 10^10^ Pa. *b* is the magnitude of the Burgers vector for dislocations, specified as 2.86 × 10^−10^ m [28]. *d* represents the diameter of the reinforcement particles, set to 1.75 × 10^−5^ m. *f* is the volume fraction of the reinforcement particles, set to 0.3.

Hall–Petch $\Delta \sigma_{Hall}$ [29]:

(2) $$\Delta \sigma_{Hall}=k_{y}\left(\frac{1}{\sqrt{D_{C}}}-\frac{1}{\sqrt{D_{0}}}\right)$$

*k_y_* is a material-dependent constant, known as the Hall–Petch slope, which characterizes the influence of grain size on yield strength, given as 0.13 MPa·m^1/2^ [29]. *D_C_* and *D*_0_ represent the grain sizes of the composite and the unreinforced material, given as 5 × 10^−6^ m and 1 × 10^−5^ m [30,31,32].

Taylor strengthening $\Delta \sigma_{Taylor}$ [29]:

(3) $$\Delta \sigma_{Taylor}=M\beta G_{m}b\sqrt{\rho }$$

*M* represents the Taylor factor, given as 3.06 [33,34]. *β* is a geometric constant, given as 1.25 [35,36]. *ρ* denotes the dislocation density, induced either by the elastic modulus mismatch *ρ_EM_* or by the thermal expansion coefficient mismatch *ρ_CTE_*, calculated below:

(4) $$\rho =\rho_{EM}+\rho_{CTE}$$

(5) $$\rho_{EM}=\frac{4\sigma_{0}f}{bdG_{m}}$$

(6) $$\rho_{CTE}=\frac{A\Delta \partial \Delta Tf}{bd\left(1-f\right)}$$

*A* is the particle shape constant, given as 12 for equiaxed particles [37]. Δα represents the difference in thermal expansion coefficients between B_4_C and 2024Al, calculated as 16.6 × 10^−6^/K. Δ*T* denotes the maximum temperature difference experienced during quenching, calculated as 470 K. $\sigma_{o}$ is the yield strength of the matrix, valued at 370 MPa.

Load transfer $\Delta \sigma_{Load}$ [38]:

(7) $$\Delta \sigma_{Load}=\frac{\left(l+t\right)s}{4l}\sigma_{o}f$$

*l* and *t* denote the lengths of B_4_C particles in the directions perpendicular and parallel to the applied stress, respectively. For equiaxed particles, *l* and *t* are equal. *s* represents the aspect ratio of the particle dimensions in the two directions and is set to 1 for equiaxed particles.

**Figure 4:**
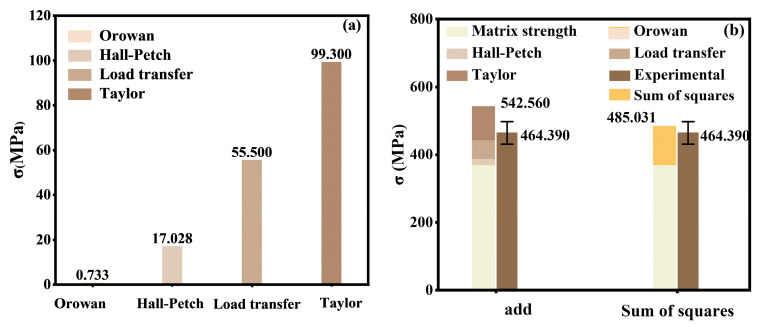
Strengthening mechanisms of the 30 vol.% B_4_C/2024Al composite. (**a**) Calculated contributions of different strengthening mechanisms to the yield strength enhancement. (**b**) Comparison of the linear superposition and the sum of squares method for predicting yield strength.

As shown in Figure 4a, the contributions of different strengthening mechanisms to the yield strength enhancement were ranked as follows: Taylor > load transfer > Hall–Petch > Orowan. Among these, the Taylor and load transfer mechanisms accounted for 57.5% and 32.1% of the total enhancement, respectively, while the remaining mechanisms collectively contributed only 10.4%. The incorporation of B_4_C increased dislocation density, enhanced dislocation interactions and hindrance effects, and provided pinning sites that restricted dislocation motion, leading to a significant contribution from the Taylor strengthening mechanism [39]. Additionally, the stronger and more abundant B_4_C particles carried a portion of the applied load, resulting in additional strengthening through the load transfer mechanism. The high dislocation density not only contributed significantly to the enhancement but also explained the pronounced strain rate strengthening and temperature softening behavior. For instance, with increasing loading rates, dislocation density rises, restricting dislocation motion, and thereby leading to strain rate strengthening.

The coupling effects between different strengthening mechanisms on the yield strength *σ_s_* of 30 vol.% B_4_C/2024Al were calculated using linear superposition and the sum of squares methods (Equations (8) and (9)), with the final results shown in Figure 4b. It can be observed that the sum of squares method produces estimates that more closely match the experimental results.

(8) $$\sigma_{s}=\sigma_{O}+\Delta \sigma_{Or\mathrm{owan}}+\Delta \sigma_{Hall}+\Delta \sigma_{T\mathrm{aylor}}+\Delta \sigma_{Load}$$

(9) $$\sigma_{s}=\sigma_{O}+\sqrt{{\left(\Delta \sigma_{Or\mathrm{owan}}\right)}^{2}+{\left(\Delta \sigma_{Hall}\right)}^{2}+{\left(\Delta \sigma_{T\mathrm{aylor}}\right)}^{2}+{\left(\Delta \sigma_{Load}\right)}^{2}}$$

### 3.3. Damage Mechanisms

**Figure 5:**
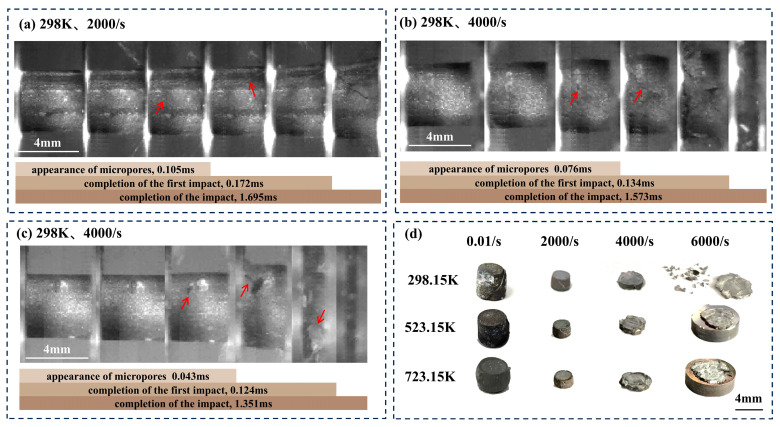
Damage progression and macro-morphology of the 30 vol.% B_4_C/2024Al composite. (**a**–**c**) High-speed images of dynamic compression at 298.15 K and strain rates of 2000/s, 4000/s, and 6000/s. (**d**) Macro-damage morphology of recovered specimens after compression.

Figure 5a–c illustrate the damage progression of the 30 vol.% B_4_C/2024Al composite under dynamic loading, recorded through high-speed imaging. Initially, the composite underwent significant axial compression with negligible lateral deformation and no visible pores or cracks, which is termed as the small plastic deformation stage. At this stage, stress was primarily concentrated along the axial direction [40,41]. As compression progressed, lateral deformation increased, and the stress state gradually transitioned from axial to triaxial. This transition led to local stress concentrations that promoted the nucleation of pores, which eventually evolved into cracks. This is referred to as the crack initiation and propagation stage. During this process, strain softening dominated, reducing load-bearing capacity and redistributing internal stresses. As the strain rate increased, crack initiation and propagation intensified, and both compression and lateral deformation were amplified. Particularly, when the strain rate reached 6000/s, the higher incident energy increased the intensity of reflected tensile waves at the edges of the sample, resulting in edge fragmentation. Finally, due to the inertia effect and the repeated transmission and reflection of stress waves within the rod, the sample experienced multiple impacts. This is identified as the inertial impact stage. At this stage, the sample experienced increased compression and intensified edge fragmentation, ultimately showing a recovered morphology.

As shown in Figure 5d, elevated temperature increased both the degree of compression and the number of cracks in the composite. As the temperature increased, the yield strength and shear strength decreased significantly, making it more prone to compression. However, due to the coupling of strain rate, the temperature softening was partially offset by strain rate strengthening. Consequently, at a strain rate of 0.01/s, the recovered sample exhibited reduced height and increased thickness compared to higher strain rates. Elevated temperature intensified plastic deformation, reducing the resistance to crack propagation. As a result, the recovered samples developed more cracks.

**Figure 6:**
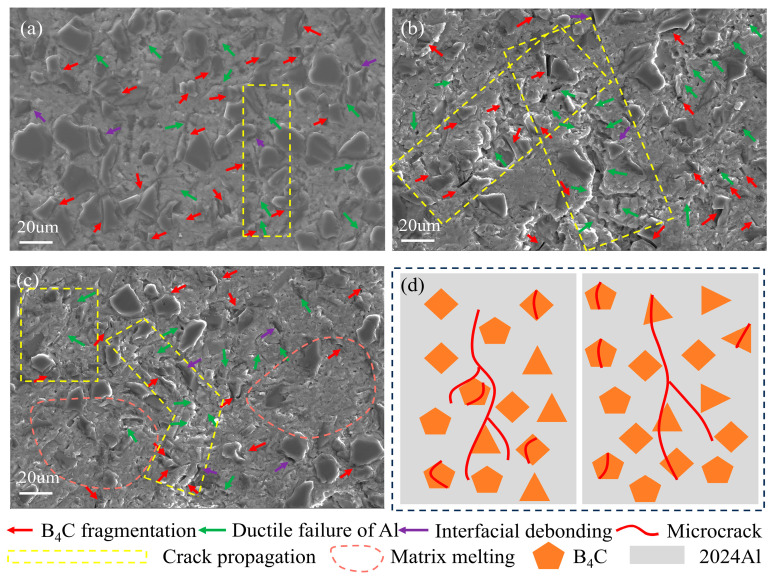
Damage modes of recovered specimens. (**a**) 0.01/s, 298.15 K. (**b**) 6000/s, 298.15 K. (**c**) 0.01/s, 723.15 K. (**d**) Two microcrack development modes.

The damage mechanisms were analyzed based on SEM images of the internal microstructure of the recovered samples. At 0.01/s and 298.15 K (Figure 6a), the B_4_C particles exhibited significant fracture (red arrows), while the matrix displayed micropores and microcracks (red arrows), with minimal interfacial debonding (purple arrows). A crack formed by matrix microcracks and interfacial debonding was observed in the yellow region. At 6000/s and 298.15 K (Figure 6b), with the increase in strain rate, matrix damage became more pronounced, and additional microcracks (green arrows) were observed. Another crack, composed of ceramic particle fracture and matrix microcracks, was found in the yellow region. Additionally, at 0.01/s and 723.15 K, further damage mechanisms were observed, including rough and uneven regions due to matrix melting at elevated temperatures (pink region).

These damage mechanisms are consistent with those reported in the literature [20,21,42]. Particle fracture and matrix damage were identified as the dominant damage modes of the 30 vol.% B4C/2024Al composite. Analysis of the yellow regions in Figure 6d revealed two distinct crack development modes, one propagated through the matrix and interface and the other traversed the ceramic particles and matrix. These are consistent with the results in other MMCs [21,43].

## 4. MJC Model & Complete Failure Criterion

### 4.1. MJC Model

According to Section 3.1, strain softening was identified as a critical stage in the stress–strain curves of the 30 vol.% B_4_C/2024Al composite. Our previous research revealed an exponential relationship between this softening and strain [44]. To accurately capture this behavior, the $C\varepsilon^{q}$ was incorporated into the strain-hardening term of the JC constitutive equation. Additionally, considering the coupled temperature and strain rate, the MJC constitutive equation was proposed as follows:

(10) $$\sigma_{y}=\left(A+B\varepsilon^{p}+C\varepsilon^{q}\right)f\left(\dot{\varepsilon },T\right)g\left(\dot{\varepsilon },T\right)$$

where *σ_y_* represents the flow stress, MPa. *A* denotes the yield strength, MPa. *B* and *p* are coefficients related to strain hardening; *B* quantifies the flow stress response to strain during strain hardening (strain hardening modulus, MPa), while *p* governs the nonlinearity. Similarly, *C* and *q* are coefficients associated with strain softening; *C* characterizes the flow stress response to strain during strain softening (strain softening modulus, MPa), and *q* controls the nonlinearity. The functions *f* and *g* capture the effects of strain rate and temperature on flow stress, respectively.

#### 4.1.1. Determination of *f* and *g*

Initially, the coupling was assessed using the temperature sensitivity index *s* and the strain rate sensitivity coefficient *m*. The calculation formulas were given in Equations (11) and (12) [45]. By plotting the logarithm of yield stress against the reciprocal of temperature, the slope was used to determine *s* and *m* was calculated through a similar approach.

(11) $$s=\frac{\partial ln(\sigma s)}{\partial (1/T)}$$

(12) $$m=\frac{\partial ln\sigma s}{\partial ln\dot{\varepsilon }}$$

**Figure 7:**
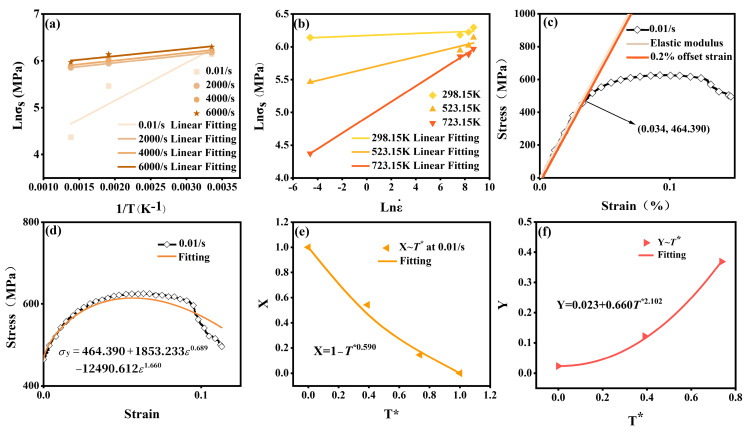
Construction and parameter calibration of the MJC constitutive equation. (**a**,**b**) Evaluation of the coupled effects of temperature and strain rate on flow stress, and determination of the temperature sensitivity coefficient ***s****,* and the strain rate sensitivity coefficient *m*. (**c**) Calibration of the yield strength parameter *A* under 0.01/s and 298.15 K. (**d**) Calibration of parameters *B*, *C*, *p*, and *q*. (**e**) Calibration of parameter *n*. (**f**) Calibration of parameters *C*_1_, *C*_2_, and *C*_3_.

A linear relationship between ln *σ_s_* and 1/T is observed in Figure 7a. At a strain rate of 0.01/s, *s* was calculated to be 806.871. As the strain rate increased to between 2000/s and 6000/s, *s* decreased and stabilized in the range of 154.006 to 165.149. These results indicated a coupling between temperature and strain rate within a specific strain rate range. To simplify the computation, the temperature softening behavior followed the JC model, as defined in Equation (13), The dimensionless temperature T* was calculated according to Equation (14), where *T_r_* denotes the reference temperature (298.15 K), *T_m_* is the melting temperature of 2024Al (875 K), and *n* represents the temperature softening index.

(13) $$g\left(T\right)=1-{\left(T^{*}\right)}^{n}$$

(14) $$T^{*}=\frac{T-Tr}{T_{m}-Tr}$$

Similarly, Figure 7b shows a linear relationship between ln *σ_s_* and $\ln\dot{\varepsilon }$. Notably, *m* exhibited a continuous increase with rising temperature, reflecting the persistent impact of temperature increase on *σ_s_* during strain rate strengthening. Therefore, the coupling of temperature and strain rate was systematically incorporated into *f*. Based on the natural logarithmic formulation of the JC constitutive equation, the final expression for *f* was given as Equation (15), where *C*_1_ is the material’s strain rate strengthening coefficient. *C*_2_ characterizes the temperature’s influence in the coupling, while *C*_3_ adjusts its nonlinearity.

$\dot{\varepsilon }$ represents the actual strain rate and $\dot{\varepsilon_{0}}$ is the reference strain rate (0.01/s).

(15) $$f\left(\dot{\varepsilon },T\right)=1+\left(C_{1}+C_{2}T^{*C_{3}}\right)\ln\left({\dot{\varepsilon }}^{*}\right)$$

(16) $${\dot{\varepsilon }}^{*}=\dot{\varepsilon }\dot{/\varepsilon_{0}}$$

Ultimately, the general form of the MJC constitutive equation was as follows:

(17) $$\sigma_{y}=\left(A+B\varepsilon^{p}+C\varepsilon^{q}\right)\left(1+\left(C_{1}+C_{2}T^{*C_{3}}\right)\ln\left({\dot{\varepsilon }}^{*}\right)\right)\left(1-T^{*n}\right)$$

#### 4.1.2. Calibration of Parameters *A*, *B*, *C*, *p*, and *q*

At the reference strain rate and temperature, Equation (17) was to the first term. As shown in Figure 7c, the parameter *A*, representing the yield strength, was determined as 464.390 MPa from the 0.2% offset strain. To avoid multiple solutions, parameters were constrained within ranges based on existing studies, and the fitting process was further optimized by maximizing the coefficient of determination (R^2^), which reached 0.974 [46,47]. The final fitting results, shown in Figure 7d, showed that *B* and *C* were 1853.233 MPa and −12490.612 MPa, and *q* and *p* were 0.689 and 1.660.

#### 4.1.3. Calibration of Parameter *n*

At the reference strain rate, the dimensionless stress ratio *X* was defined and calculated according to Equation (18), with *X* representing the mean value of the experimental results at each temperature. The fitting result, shown in Figure 7e, was determined as 0.590.

(18) $$X=\left(1-T^{{*}^{n}}\right)=\frac{\sigma_{y}}{\left(464.390+1853.233\varepsilon^{0.689}-12490.612\varepsilon^{1.660}\right)}$$

#### 4.1.4. Determination of Parameters *C*_1_, *C*_2_, and *C*_3_

Similarly, the dimensionless quantity *Y* was defined and calculated according to Equation (19). The results, shown in Figure 7f, revealed that *C*_1_, *C*_2_, and *C*_3_ were obtained as 0.023, 0.660, and 2.102.

(19) $$Y=C_{1}+C_{2}T^{*C_{3}}=\frac{\sigma_{y}/\left[\left(464.390+1853.233\varepsilon^{0.689}-12490.612\varepsilon^{1.660}\right)\left(1-T^{*0.590}\right)\right]-1}{\ln\left({\dot{\varepsilon }}^{*}\right)}$$

The final parameterized MJC constitutive equation was as follows:

(20) $$\sigma_{y}=\left(464.390+1853.233\varepsilon^{0.689}-12490.612\varepsilon^{1.660}\right)\left(1+\left(0.023+0.660T^{*2.102}\right)\ln\left({\dot{\varepsilon }}^{*}\right)\right)\left(1-T^{*0.590}\right)$$

### 4.2. Complete Failure Criterion

As discussed in Section 3.1, the failure behavior of the 30 vol.% B_4_C/2024Al composite was closely associated with stress triaxiality, making the JC failure equation suitable for characterizing its influence on failure [48]. However, in compression tests, the specimen is subjected to uniaxial loading and does not experience a fully triaxial stress state, which makes a single compression test insufficient to comprehensively predict the material’s failure behavior [49]. In this study, the MJC model incorporates strain softening, and the strain energy density (*W_D_*) was further proposed as the criterion for complete failure. These approaches provided a more precise and comprehensive determination of the composite’s failure.

The general form of the complete failure criterion, informed by the structure of the JC failure equation and the coupling in the MJC model, was established and presented in Equation (21). In this equation, *W_O_* represents the basic strain energy density at the reference strain rate and temperature. *D*_1_ denotes the strain rate strengthening coefficient. *D*_2_ captures the temperature effect on the coupling during strengthening, while *D*_3_ adjusts the nonlinearity. *D*_4_ governs the overall nonlinearity of the strain rate strengthening. *D*_5_ represents the temperature softening coefficient, and *D*_6_ modulates its nonlinearity.

(21) $$W_{D}=W_{O}\left(1+\left(D_{1}+D_{2}T^{*D_{3}}\right){\left(\ln\left({\dot{\varepsilon }}^{*}\right)/10\right)}^{D_{4}}\right)\left(1-D_{5}T^{*D_{6}}\right)$$

#### 4.2.1. Calibration of Parameters *D*_5_ and *D*_6_

**Figure 8:**
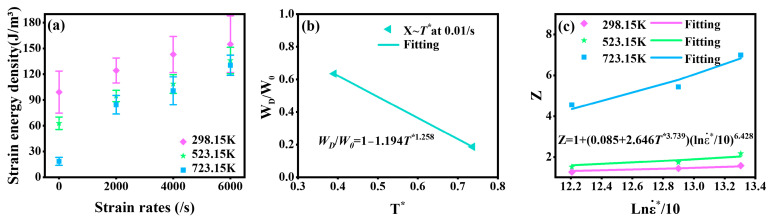
Calibration of parameters for the complete failure equation. (**a**) Strain energy density under experimental conditions. (**b**) Calibration of parameters *D*_5_ and *D*_6_. (**c**) Calibration of parameters *D*_1_, *D*_2_, *D*_3_, and *D*_4_.

By integrating the curve from Figure 3c–f, *W_D_* under various experimental conditions was obtained, as shown in Figure 8a. The key parameter *W_O_* was calculated as 99.15. To calibrate the coefficients related to temperature softening, *W_D_* under the reference strain rate was employed to establish a relationship with T*, as illustrated in Figure 8b. The final values of *D*_5_ and *D*_6_ were determined to be 1.194 and 1.258.

#### 4.2.2. Calibration of Parameters *D*_1_, *D*_2_, *D*_3_, and *D*_4_

Equation (22) was developed to describe the relationship between $\ln\left({\dot{\varepsilon }}^{*}\right)/10$ and *Z* at different temperatures. As shown in Figure 8c, the variation in the slope of the fitted curves with temperature confirmed the coupling effects of temperature and strain rate on *W_D_*. The final values of *D*_1_, *D*_2_, *D*_3_, and *D*_4_ were determined to be 0.085, 2.646, 3.739, and 6.428.

(22) $$Z=\frac{W_{D}}{W_{O}\left(1-D_{5}T^{*D_{6}}\right)}=1+\left(D_{1}+D_{2}T^{*D_{3}}\right){\left(\ln\left({\dot{\varepsilon }}^{*}\right)/10\right)}^{D_{4}}$$

(23) $$W_{D}=99.15\left(1+\left(0.085+2.646T^{*3.739}\right){\left(\ln\left({\dot{\varepsilon }}^{*}\right)/10\right)}^{6.428}\right)\left(1-1.194T^{*1.258}\right)$$

### 4.3. Prediction Performance of the MJC

**Figure 9:**
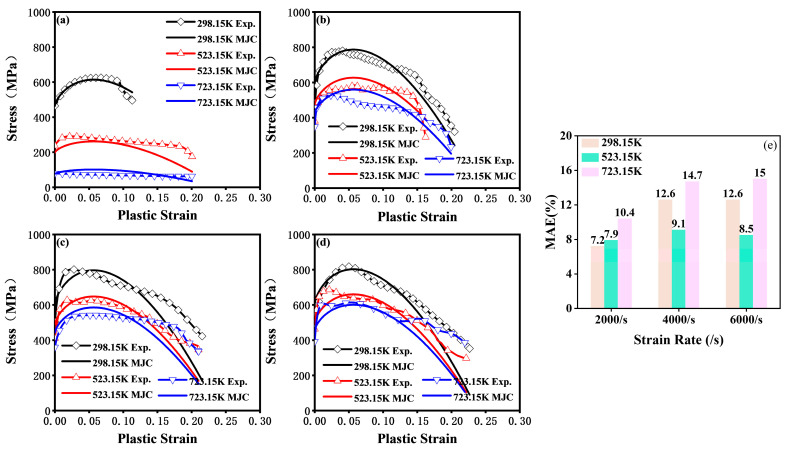
Fitting results of the MJC constitutive equation. (**a**–**d**) Comparison of the stress–strain relationships predicted by the MJC with experimental data at strain rates of 0.01/s, 2000/s, 4000/s, and 6000/s. (**e**) Mean absolute error of the MJC predictions under dynamic loading conditions.

As shown in Figure 9a–d, the predictions of the MJC model closely aligned with the stress–strain curves obtained from experiments, effectively capturing key characteristics of plastic deformation, including strain hardening, strain softening, strain rate strengthening, and temperature softening. Further error analysis, as shown in Figure 9e, revealed that at higher temperatures and strain rates, the fluctuation of the stress–strain curve intensified due to factors such as variations in the local microstructural characteristics of the material during testing, leading to an increase in the mean absolute error (MAE). However, it completely remained below 15% under dynamic loading.

## 5. Numerical Simulation Results and Discussion

**Figure 10:**
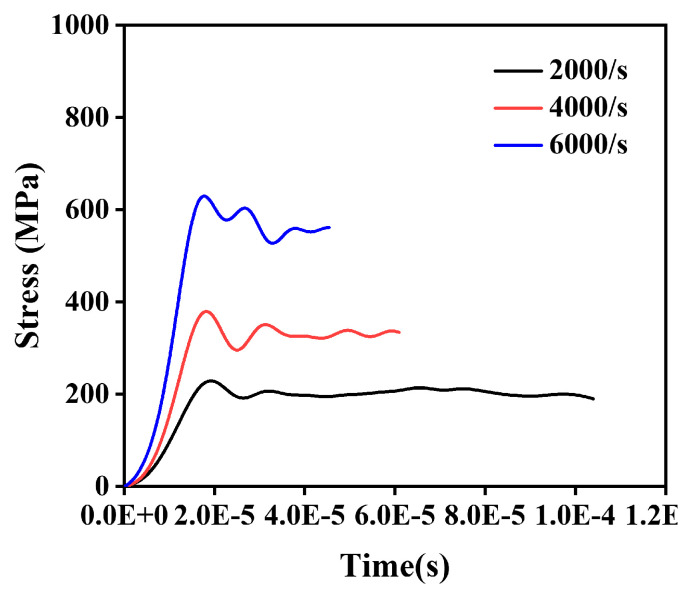
Stress–time loading curves at different strain rates.

**Figure 11:**
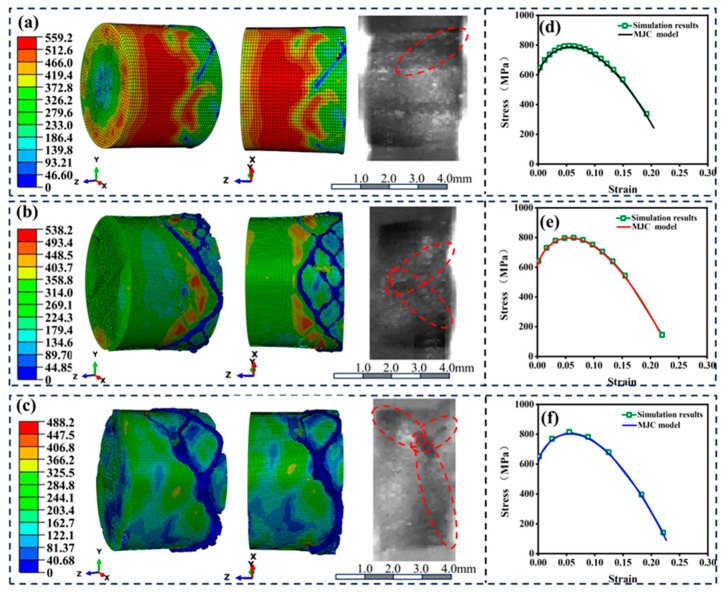
Validation of VUHARD subroutine in SHTB finite element analysis. (**a**–**c**) Damage morphologies from finite element analysis and experiments at strain rates of 2000/s, 4000/s, and 6000/s. (**d**–**f**) Comparison of the stress–strain curves from finite element analysis and experiments at strain rates of 2000/s, 4000/s, and 6000/s.

Based on the stress–time loading curves shown in Figure 10, SHPB simulations were conducted at different strain rates, with the results presented in Figure 11. Figure 11a shows the axial compression of the 30 vol.% B_4_C/2024Al composite at a strain rate of 2000/s. The composite exhibited significant lateral plastic expansion, and oblique shear cracks were observed on the side. As the applied stress increased and the strain rate was raised to 4000/s, both the compression and lateral deformation of the composite increased, with the oblique shear cracks connecting (Figure 11b). When the strain rate increased to 6000/s, in addition to further compression and oblique crack propagation, localized fragmentation at the edges of the composite was observed (Figure 11c). These observations were in excellent agreement with the experimental results, as confirmed by the high-speed camera images captured at the completion of the first impact test. Furthermore, the stress–strain curves extracted from randomly selected elements within the model at their respective strain rates demonstrated strong consistency with the results obtained from the MJC model, as shown in Figure 11d–f.

These results demonstrate that the finite element analysis, combined with the VUHARD subroutine, successfully simulated the axial compression and lateral expansion plastic deformation behaviors of the composite material. By controlling the deletion of complete failed elements, the model accurately captured phenomena such as oblique shear cracking and localized fragmentation. Under dynamic impact conditions, the subroutine showed excellent applicability.

## 6. Conclusions

This study investigates the dynamic compressive behavior of a 30 vol.% B_4_C/2024Al composite across a wide range of strain rates (0.01–6000/s) and temperatures (298.15 K–723.15 K). Based on experimental results, the modified Johnson–Cook (MJC) constitutive model and a complete failure criterion were proposed and implemented in the VUHARD subroutine for finite element analysis. The key conclusions are as follows:

1. The 30 vol.% B_4_C/2024Al composite exhibited both strain hardening and strain softening behaviors under different experimental conditions. Significant strain rate strengthening and temperature softening were observed, with a notable coupling between these two effects. A detailed analysis of the composite’s yield strength enhancement revealed that the Taylor and load transfer mechanisms were the dominant contributors, accounting for 89.6% of the total strengthening. Damage progression under dynamic loading occurred in stages, beginning with minor plastic deformation, followed by crack initiation and propagation, and culminating in inertial impact. Particle fracture and matrix damage were identified as the primary damage mechanisms.

2. The MJC model, which incorporates strain softening and the coupling effects of strain rate and temperature on flow stress, was developed. Additionally, a complete failure criterion based on strain energy density (W_D_) was proposed. The MJC model demonstrated high accuracy, with a mean absolute error (MAE) of less than 15% between its predictions and the experimental data under dynamic loading. The MJC model and the complete failure criterion are expressed as follows:

$$\sigma_{y}=\left(464.390+1853.233\varepsilon^{0.689}-12490.612\varepsilon^{1.660}\right)\left(1+\left(0.023+0.660T^{*2.102}\right)\ln\left({\dot{\varepsilon }}^{*}\right)\right)\left(1-T^{*0.590}\right)$$

$$W_{D}=99.15\left(1+\left(0.085+2.646T^{*3.739}\right){\left(\ln\left({\dot{\varepsilon }}^{*}\right)/10\right)}^{6.428}\right)\left(1-1.194T^{*1.258}\right)$$

3. Using the VUHARD subroutine, ABAQUS simulations of the split Hopkinson pressure bar (SHPB) accurately captured the composite’s plastic deformation, crack propagation, and localized fragmentation under dynamic loading at strain rates of 2000/s, 4000/s, and 6000/s. The simulation results showed excellent agreement with the experimental findings.

4. This study presents a constitutive model and a complete failure criterion that effectively describes the plastic deformation and failure processes of ceramic-reinforced metal matrix composites. Future work will extend the model to composites with varying volume fractions and validate it through a series of ballistic tests, further enhancing its applicability and robustness in practical scenarios.

## Data Availability

The data presented in this study are available on request from the corresponding author.
